# Limbal epithelial stem cell activity and corneal epithelial cell cycle parameters in adult and aging mice

**DOI:** 10.1016/j.scr.2018.11.001

**Published:** 2018-12

**Authors:** Nada Sagga, Lucia Kuffová, Neil Vargesson, Lynda Erskine, J. Martin Collinson

**Affiliations:** a School of Medicine, Medical Sciences and Nutrition, University of Aberdeen, Institute of Medical Sciences, Foresterhill, Aberdeen AB25 2ZD, United Kingdom; b Department of Ophthalmology, NHS Grampian, Aberdeen, UK

**Keywords:** Cornea, Wounding, Limbal stem cells, Proliferation, Label-retaining cells

## Abstract

Limbal epithelial stem cells (LESCs) are believed to be responsible for corneal epithelial maintenance and repair after injury, but their activity has never been properly quantified in aging or wounded eyes. In this study, labelling with thymidine analogues, 5-iodo-2′-deoxyuridine (IdU), 5-chloro-2′-deoxyuridine (CldU) and 5-ethynyl-2′-deoxyuridine (EdU), was used to estimate cell-cycle time of the corneal and limbal epithelia in wild-type eyes, comparing aging (12 months) and young adult (8 week) mice. In C57BL/6 mice, cells cycled significantly faster in the central corneal epithelium of aging eyes (3.24 ± 0.2 days) compared to 10 week old mice (4.97 ± 0.5 days). Long-term labelling with IdU was used to detect slow-cycling stem cells, followed by CldU or EdU labelling to quantify the proliferative dynamics of LESCs during corneal wound healing. In unwounded eyes, 4.52 ± 1.4% of LESCs were shown to enter S phase in a 24 h period and were estimated to divide every 2–3 weeks. Within 24 h of corneal injury this rose significantly to 32.8 ± 10.0% of stem cells indicating a seven-fold increase in activation. In contrast, no comparable increase in LESC activation was observed in aging mice after wounding. In the 24–48 h period after wounding in young adults, LESC activation continued to increase (86.5 ± 8.2% of label-retaining cells in wounded eye were in S-phase) but surprisingly, 46.0 ± 9.4% of LESCs were observed to reenter S-phase in the contralateral unwounded eye. These data imply an unsuspected systemic effect of corneal wounding on LESC activation suggesting that injury to one eye elicits a regenerative response in both.

## Highlights

• Corneal wounding causes a seven-fold increase in the number of limbal epithelial stem cells in mitosis, 24 h after injury.
• This stem cell response to injury does not occur in aging animals.
• 24–48 h after wounding, nearly 90% of limbal epithelial stem cells are in mitosis.
• Wounding to one cornea elicits a systemic stem cell response in the other cornea after 24 h.

## Graphical abstract

**Figure:**
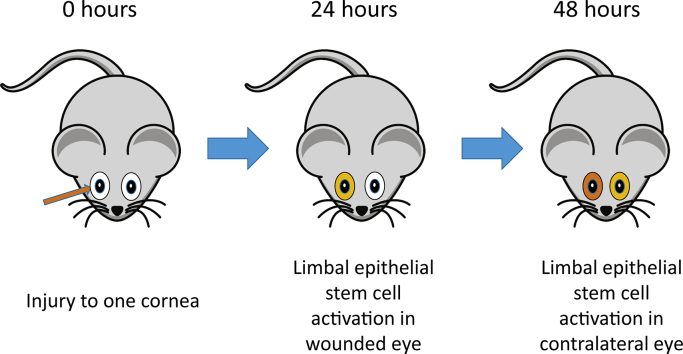

## 1 Introduction

Maintenance of the ocular surface epithelium is required for normal vision. The stratified corneal epithelium is subject to constant abrasion, e.g. by eye blinking; and apical squamous cells are continuously lost from the uppermost layer (Forrester et al., 2002). Cell division is restricted to the basal layer, with postmitotic differentiated cells losing contact with the basement membrane and moving apically to maintain normal epithelial thickness (Lehrer et al., 1998; Dua and Azuara-Blanco, 2000; Yoon et al., 2014). In addition, the corneal renewal process is believed to require a peripheral ring of limbal epithelial stem cells (LESCs) at the border of the corneal and conjunctival epithelia. These are a slow-cycling, small, undifferentiated cells which divide to produce proliferative ‘transit amplifying’ basal epithelial cells (TACs) that migrate and divide further to repopulate the basal corneal epithelium during normal homeostasis and replace those cells desquamated or lost by abrasion (Schermer et al., 1986; Cotsarelis et al., 1989; Daniels et al., 2001; Collinson et al., 2002; Nagasaki and Zhao, 2003; O'Sullivan and Clynes, 2007; Osei-Bempong et al., 2013; Di Girolamo et al., 2015; Dorà et al., 2015; Kasetti et al., 2016).

Several lines of evidence have shown that the corneal epithelium itself has efficient regenerative ability (Huang and Tseng, 1991; Majo et al., 2008; Dua et al., 2009; Kawakita et al., 2011). Newborn transit amplifying cells produced from division of an LESC have high, but not indefinite, proliferative potential (Mort et al., 2012). TACs divide more rapidly than stem cells but undergo only a limited number of cell divisions before terminally differentiating (Kruse, 1994; Ren and Wilson, 1996; Pellegrini et al., 1999). Hence high levels of mitosis in the basal epithelial layer of the cornea contribute very significantly to the regenerative potential of the cornea but must be regulated such that rate of cell replacement equals cell loss – one possibility is that cell cycle rate slows as cells progress through successive mitoses to terminal differentiation (Ramaesh et al., 2003; Mort et al., 2012). The complete turnover and replacement of lost corneal epithelial cells has been estimated to take 2 weeks in mice (Buck, 1985; Cenedella and Fleschner, 1990; Meyer-Blazejewska et al., 2011).

Biochemically, distinguishing LESCs from TAC progenitors is a challenging task. There has been a great deal of controversy over the investigation of LESC markers based on their morphology, clonogenicity and phenotype (Utheim, 2013). However, the slow-cycling, label-retaining phenotype of stem cells has been widely accepted and exploited to mark their location in the limbus (Cotsarelis et al., 1989; Lavker and Sun, 2003; Lavker et al., 2004). Label-retention assays require long-term incorporation of DNA labelling reagents such as thymidine analogues into the DNA of dividing cells. Such a prolonged exposure of cells to the label (typically several days or weeks) ensures uptake by most proliferative cells (Yennek and Tajbakhsh, 2013). A long subsequent chase period, however, allows rapidly dividing cells (e.g. TACs) to dilute the label while slow-cycling cells to retain it, hence, the terminology ‘label-retention’. This approach has been used to identify slow-cycling cells in the corneal epithelium, as well as in other epithelial tissues such as hair follicles (Cotsarelis et al., 1990), mammary gland (Zeps et al., 1998) and intestinal crypts (Potten et al., 2002). LESCs were identified as slow cycling on the basis of their ability to retain tritiated thymidine (^3^H-TdR), over a long period (Cotsarelis et al., 1989), though how often they divide remains uncertain. In the event of injury or disease causing significant corneal epithelial cell loss, LESCs are believed to divide more frequently to re-establish homeostasis, but although this assumption underlies much of our understanding of ocular surface regeneration the published evidence is limited. Cotsarelis et al. (1989) showed an increase in limbal epithelial cell proliferation after injury but did not show this was due to the previously slow-cycling stem cells. Lehrer et al. (1998), using 5-bromo-2′-deoxyuridine (BrdU)/^3^H-thymidine double-labelling showed that many label-retaining limbal epithelial cells can proliferate 24 h after injury, but did not accurately quantify the result or report on the unoperated contralateral eyes. Hence one of the crucial assumptions underlying clinical concepts of corneal maintenance is not strongly supported.

Function of LESCs is clinically important. Limbal stem cell deficiency, caused for example by physical injury or alkali burn, is characterized by thinning of the corneal epithelium, conjunctivalisation, inflammation, pannus and subsequent corneal blindness. Corneal epithelial changes secondary to infections, e.g. herpes simplex virus or *Chlamydia trachomatis* (trachoma), is one of the leading causes of acquired blindness worldwide.

Like most of the tissues in the body, aging has been found to cause structural and functional changes in corneas (Gipson, 2013). Age-related changes include loss of corneal sensitivity (Roszkowska et al., 2004) possibly due to the decrease in nerve density in the sub-basal epithelial nerve plexus (Niederer et al., 2007). Reduction in corneal endothelial cell density is also well documented with aging (Hoppenreijs et al., 1994; Blake et al., 1997). Epithelial thickness exhibits gradual deterioration in human limbal epithelia and peripheral corneas with aging, but not the central cornea (Cerulli and Missiroli, 2008; Yang et al., 2014). Although these studies have shown that increasing age can alter the structure of the corneal epithelium, very little is known about the effect of aging on LESC-derived progenitor proliferation, or corneal renewal. Conventional dogma would predict a loss of stem cell activity with age, though no study has assessed this for LESCs.

This study has investigated quantitatively for the first time the activation and proliferation rate of slow-cycling LESCs after corneal damage and investigated how these can be affected by aging. We show how the cell-cycle kinetics of TACs in corneal epithelium changes with aging and show that injury to one eye may activate LESCs in the contralateral unwounded eye.

## 2 Material and methods

### 2.1 Ethics statement

Mice were housed in the Medical Research Facility at the University of Aberdeen, where all animal care and welfare procedures and ethical regulations were followed. All experimental protocols and surgery were authorized by the Home Office in accordance to the Animals (Scientific Procedures) Act 1986.

### 2.2 Cell culture

A human corneal epithelial cell line (HCE-S) (Notara and Daniels, 2010) was maintained in DMEM/F12 culture medium with 10% fetal calf serum. For S-phase labelling, 5-iodo-2′-deoxyuridine (‘IdU’ – Sigma I7125) or 5-ethynyl-2′-deoxyuridine (EdU – ThermoFisher E10187) was added to cells in 24 well plates to a final concentration of 10 μg/ml.

### 2.3 Experimental mice

C57BL/6 mice were commercially sourced (Charles River, UK) at 8 weeks and 12-month-old to compare cell cycling kinetics in corneal tissues between ages. For LESC activity and proliferation studies, adult (8 weeks old at start of experiment) and aging (8 months old at start of experiment) C57BL/6 mice were used.

### 2.4 Circulation time of IdU solution in mice

To identify the minimum time for IdU solution to circulate and label corneal and limbal epithelial cells, mice were intraperitoneally injected with a single dose of IdU (2 mg/ml in saline) and allowed to circulate for 5 min, 15 min or 2 h. Mice were then humanely culled and within a few seconds eyes were enucleated and placed into cold 4% paraformaldehyde (PFA) fixative for immunofluorescence analysis.

### 2.5 Short-term double-pulse of IdU/CldU or IdU/EdU in mice

To identify the kinetics of proliferating TACs in the central cornea, peripheral and limbus of mice, a double pulse method was performed similar to the method introduced by Martynoga et al. (2005) to allow calculation of the duration of S-phase (*Ts*) and the length of a cell cycle (*Tc*). Each mouse received a single intraperitoneal injection of 2 mg/ml IdU followed 1.5 h later 2 mg/ml EdU for 30 min before the mice were killed. Detection of single-labelled and double-labelled IdU/EdU cells were analyzed by immunofluorescence staining as described below. The experiment revealed three populations of positive labelled cells; 1) IdU^+^ (red cells) that left the S-phase of cell cycle *(L*_cells_) during the inter-injection interval 1.5-hour (*Ti*); 2) EdU^+^ (green cells) that entered the S-phase during the 30 min after EdU injection; 3) a population of IdU^+^/EdU^+^ (red and green labelled cells) (*S*_cells_) were also found representing cells in S-phase during both phases of labelling. To determine the ratio of those populations of labelled cells, the total number of proliferating cells in the sampling area (*P*_cells_) visualized by TOPRO-3 iodide nuclear staining was counted. The length of S-phase (*Ts*) and the total cell-cycle time (*Tc*) for proliferating TACs was calculated based on the formula adopted from Martynoga et al. (2005) as follows:

$$\mathrm{Ts}=\mathrm{Ti}/\left(L_{\mathrm{cells}}/S_{\mathrm{cells}}\right)∴\mathrm{Tc}=\mathrm{Ts}/\left(S_{\mathrm{cells}}/P_{\mathrm{cells}}\right)$$

### 2.6 Long-term IdU administration and establishment of IdU-label-retention

DNA label-retention assay was used to identify slow-cycling LESCs in C57BL/6 mice. For 30 consecutive days IdU solution (1 mg/ml) was administrated to mice via drinking water, and then followed with different interval washout periods via normal drinking water for 0 to 10 weeks. IdU-label-retaining cells were visualized on both corneal wax sections and whole-mount corneal tissues by immunohistochemistry and immunofluorescence staining, respectively.

### 2.7 In vivo corneal epithelial wounding

In vivo corneal epithelial scraping procedure was performed in long-term IdU-labelled mice. In a sterile environment and under general anaesthesia, using an operating microscope (OPMI VISU 150, Zeiss, Germany), a central, circular, partial corneal epithelial cut was performed with a 1.5 mm trephine (Katina, K2–7520) in the right eye of the animal. Using a sterile surgical blade, the corneal epithelial tissue within the circled defined area was fully debrided. The contralateral left eye kept unwounded.

### 2.8 Assessing the activity of label-retaining cells during corneal epithelial wound healing

To monitor the activity and proliferative response of slow-cycling LESCs (i.e. IdU-label-retaining cells) during corneal wound healing, a short-term pulse of 5-chloro-2′-deoxyuridine (CldU) (10 mg/ml) or EdU (2 mg/ml) was given by intraperitoneal injection, either at 0 h or 24 h after epithelial wounding. The experimental design of injecting CldU or EdU after wounding is to discriminate between two populations of IdU-label-retaining cells (IdU-LRCs): those that are quiescent and those that become active and proliferative after wounding. The activation time of IdU-LRCs was analyzed by immunofluorescence through double staining nuclei IdU/CldU or IdU/EdU. EdU and CldU were functionally interchangeable and gave identical results.

### 2.9 Preparation of tissue sections and immunohistochemistry staining for IdU

#### 2.9.1 Tissue preparation

Whole eyes were fixed with 4% PFA and rinsed in PBS (3 × 10 min). Fixed eyes were gradually dehydrated in a series of increasing ethanol concentrations (70%, 85%, 95% and 100%) for 15 min each. Dehydrated eyes were cleared by xylene washes (2 × 5 min, each) then overnight in fresh xylene. Eyes were embedded in paraffin. Corneal sections (5 μm-thick) were cut and mounted on poly-l-lysine coated slides, deparaffinised in Histo-Clear (2 × 10 min, each) and rehydrated in a series of decreasing ethanol concentrations.

#### 2.9.2 Immunohistochemistry

Immunohistochemistry with 3,3′-diaminobenzidine colour endpoint was performed as described in Collinson et al. (2003). Sections were incubated with primary antibody mouse anti-BrdU (Abcam-ab8955) (1:200 dilution) overnight at 4 °C. Secondary antibody was biotinylated goat anti-mouse IgG (Vector laboratories) (1:200), at room temperature for 2 h. After colour reaction, tissues were rinsed with H_2_O and counterstained with hematoxylin for 30 s, dehydrated in a series of increasing ethanol concentrations, cleared in xylene and mounted using DPX mounting medium (Cellpath) for light microscopy imaging.

### 2.10 Whole-mount immunofluorescence staining of IdU/CldU and IdU/EdU

Dissected corneal-limbal tissues were permeabilized with cold methanol at −20 °C for 10 min and rinsed 3 times in PBS. DNA was denatured with 2 M HCL at 37 °C for 15 min, neutralised with 0.1 M sodium borate buffer (pH 8.5) at room temperature for 20 min, followed by rinses with PBS (3 × 10 min) and 2 h incubation in blocking buffer (0.3% BSA in PBS; 4% donkey serum, 0.1% Triton X-100), at room temperature. Tissues were incubated with the primary antibodies, mouse anti-IdU (ab181664) (1:250 dilution) and rat anti-CldU (ab6326) (1:250 dilution) overnight at 4 °C. On the following day, tissues were rinsed with PBS (3 × 20 min), and co-incubated with secondary antibodies, Alexa Fluor 594 goat anti-mouse IgG1 (1:200 dilution) and cross-absorbed goat anti-rat IgG (1:200 dilution) (Millipore-AP136F), for 3 h at room temperature away from light. Tissues were rinsed with PBS (3 × 20 min) and counterstained with TO-PRO-3 Iodide (T3605-Life technologies) (1:1000 dilution in PBS) for 30 min. Following additional rinses with PBS, tissues were cut into 16 sectors to lie flat and mounted on microscope slides using Vectashield mounting medium (H-1000-Vector Laboratories).

The protocol of double-immunofluorescence staining of IdU/EdU was similar to the above IdU/CldU immunofluorescence staining method except for the following: after permeabilization with cold methanol, EdU label was first detected by incubating in Click-iT® EdU reaction -Alexa 488 azide (ThermoFisher C10337) according to manufacturer's instructions. The reaction was carried out in accordance with the manufacturer's instructions, for 30 min at room temperature, protected from light. Then IdU was detected using the same steps described above. There was no cross-reactivity between this IdU antibody and EdU or CldU, shown by injecting mice with EdU or CldU only and then performing anti-IdU immunohistochemistry to confirm absence of signal.

### 2.11 Image acquisition and analysis

Images of the epithelia of central, peripheral corneas or limbal area were acquired under a fluorescence microscope (Axio Imager M2; Carl Zeiss) with a 40× objective. The limbal epithelium was defined anatomically using the criteria outlined in Douvaras et al. (2013). For each limbal or corneal peripheral tissue, eight images were taken, in which a single image represents one sector of 16 sectors in a whole-mount tissue. For central cornea, six images were taken from each whole mount tissue. Each acquired image of central, peripheral corneas or limbus shows cells labelled with either IdU/CldU/TO-PRO3 iodide or IdU/EdU/TO-PRO3 iodide. DAB staining in corneal wax sections were imaged under a bright field microscope (Nikon Eclipse E400) with objective 20 X. Labelled HCE-S cells in culture plates were imaged with an inverted fluorescence microscope (Leica DM IRB).

### 2.12 Statistical analysis

ImageJ (version 1.49 t) were used for counting labelled cells. Data were statistically analyzed using GraphPad Prism (version 5.04) and IBM SPSS statistics (version 24).

## 3 Results

### 3.1 Label-retention and cell cycle parameters in vitro

**Fig. 1:**
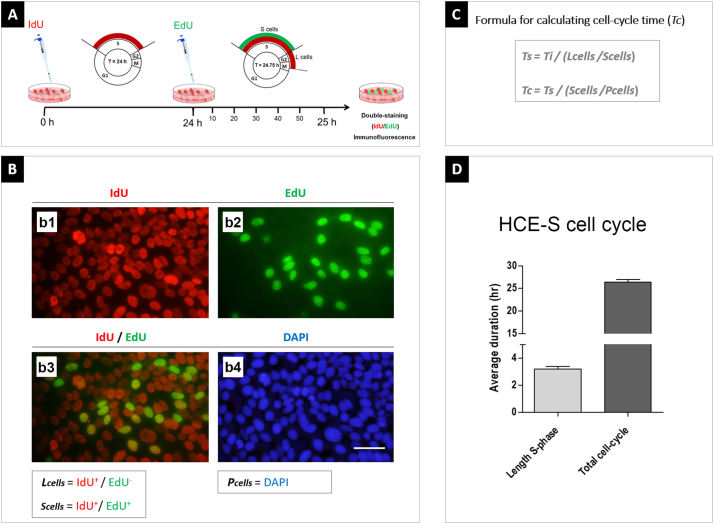
Length of S-phase and total cell cycle time for confluent HCE-S cells. (A) Methodological scheme illustrating the 24-h interval between IdU and EdU pulses (45 min) in vitro. (B) IdU-labelled cells (red, b1) and EdU-labelled cells (green, b2). IdU^+^/EdU^−^ cells are those that have left S-phase during the 24 h of IdU administration (*L*_cells_). Double-labelled IdU^+^/EdU^+^ cells represent all cells in S-phase during the experiment (*S*_cells_). DAPI (blue) cells are the total number cells, all of which are potentially proliferative, and represents *P*_cells_. (C) Formulae used to calculate S-phase length and cell cycle time. (D) Mean ± s.e.m. time (hours) of S-phase and cell cycle for confluent HCE-S cells. *Abbreviations: Tc* = *cell cycle time; Ts* = *length of S-phase; Ti* = *24 h interval when cells were exposed to IdU but not EdU; h* = *hour.* Scale bars = 50 μm.

In order to validate the protocol for measuring cell cycle time in ocular surface epithelia, confluent human corneal epithelial cells (HCE-S as described in Materials and Methods) were cultured in the presence of the thymidine analogue, IdU, for 24 h, to label all cells in S-phase, then given a 45-min pulse of a separate thymidine analogue, EdU for 45 min. Double fluorescence labelling of IdU/EdU was then performed followed by counting of double- and single - labelled cells to estimate length of S-phase (*Ts*) and total cell cycle time (*Tc*) as described in Materials and Methods. The length of S-phase for confluent HCE-S cells was calculated at 4.1 ± 0.1 h and cell cycle time at 34.3 ± 1.5 h (n = 6 replicates), consistent with the rapid-doubling phenotype of these cells (Fig. 1) (Notara and Daniels, 2010).

**Fig. 2:**
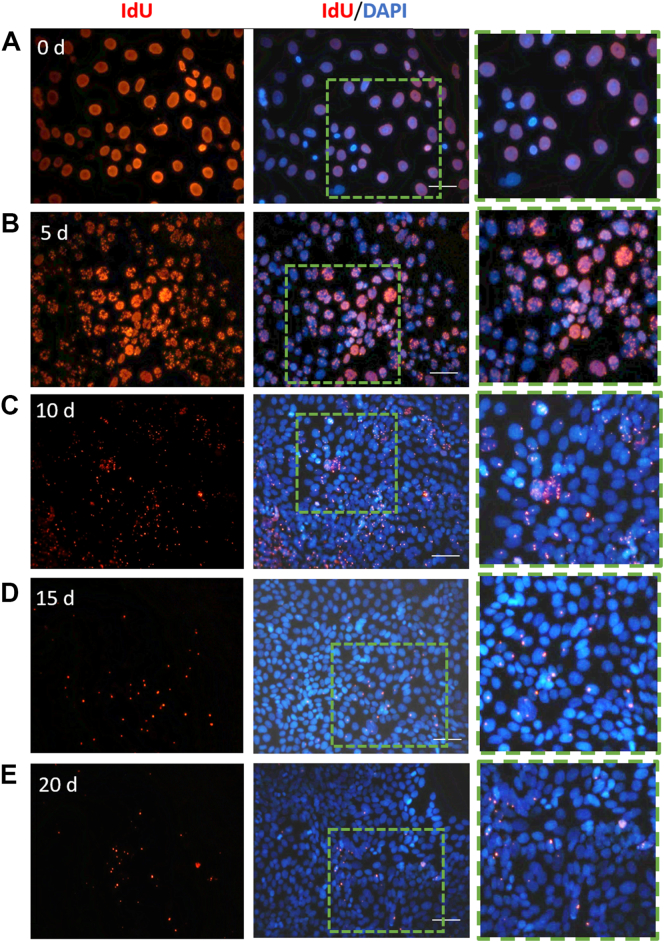
IdU-label retention in confluent HCE-S cells. (A–E) HCE-S cells labelled with IdU for 2 days then maintained in normal medium for chase periods of 0–20 days. Immunocytochemistry for IdU (red) with DAPI counterstain. IdU label starts to dilute uniformly from cells at 5 days and appears as increasingly speckled labeling. Insets in fluorescent images show the magnification of cells that are labelled with IdU. Scale bars = 30 μm. Data are representative of triplicate experiments.

The HCE-S cell line is reported to maintain stem-cell characteristics, evidenced by its colony-forming efficiency and expression of genes such as ABCG2 and ΔNP63α, considered to be markers for human limbal epithelial stem cells (Notara and Daniels, 2010). As a clonally-derived cell line, all HCE-S cells should be identical. In order to determine whether ‘label-retention’ is a genuine assay for slowly-dividing cells in a population, or an artefactual ‘tail’ of detection in otherwise uniformly dividing population of ocular surface epithelial cells, a label-retention experiment was performed on HCE-S cell culture. Confluent cells were exposed to IdU in vitro for a 48-h pulse. Cultures were then maintained in normal medium for chase periods of 0–20 days before being fixed and stained for immunohistochemistry. Representative data are presented in Fig. 2. 100% of cells were labelled by the IdU pulse at 0 days, consistent with estimated cell cycle time (above) (n = 4 cultures). IdU was shown to dilute uniformly, with speckled labelling of >99% cells at 5 days of washout (approx. 3 cell divisions). However levels of detection subsequently dropped rapidly. At 10 days (6–7 cell divisions), IdU was sporadic but still detectable, at least as isolated fluorescent spots, in 35.1% ± 1.9 of the HCE-S cells (n = 14), and in 5.8% ± 0.9 at 15 days (n = 14). Only 1.3% ± 0.2 of cells retained detectable IdU after 20 days. No outlier cells with unusually high levels of retained label were found (3 repeats, > 40,000 cells assayed). These data confirm that the property of thymidine analogue ‘label-retention’ at the ocular surface is not an assay artefact of stochastic effects in a uniform population of dividing cells.

### 3.2 Detection of IdU-labelled cells in corneal and limbal epithelia of mice

**Fig. 3:**
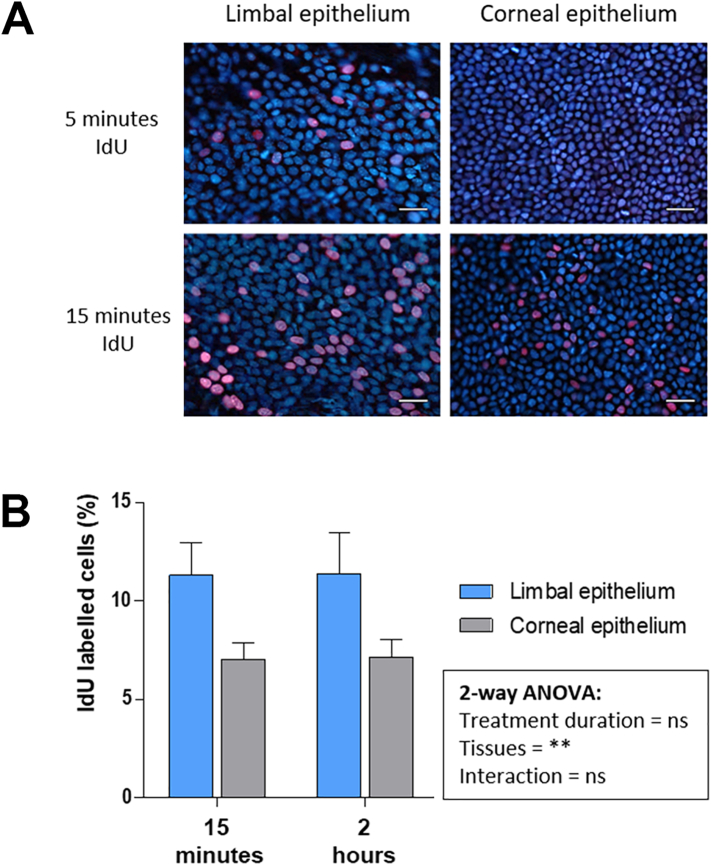
Circulation time and distribution of IdU to the limbal and corneal epithelia of mice. (A) Immunofluorescent staining of flat-mounted limbal and corneal epithelia for IdU (red) and TO-PRO-3 nuclear counterstain (blue). IdU-positive cells in limbal epithelium but not in corneal epithelium 5 min after injection. 15 min of IdU are sufficient to label both limbal and corneal epithelial cells. Scale bars = 20 μm. Cells density is higher in the corneal epithelium than in the limbus. (B) Mean (%) ± SEM of %IdU-labelled cells in the limbal and corneal epithelia 15 min (n = 6) and 2 h (n = 6) after intraperitoneal-injection. (For interpretation of the references to colour in this figure legend, the reader is referred to the web version of this article.)

To determine how quickly IdU-labelled cells can be detected in limbal and corneal epithelial cells, wild-type mice were injected with IdU solution then killed after 5 min, 15 min or 2 h. IdU-labelled cells that were in S-phase were detectable after 5 min in limbal epithelia, but not in the basal proliferative layer of the corneal epithelium. After 15 min, both limbal and basal corneal epithelial cells were labelled (Fig. 3A). Presumably, IdU was transported first to the limbus through systemic and conjunctival blood vessels, then to the avascular corneal epithelium via the aqueous humor. When the percentage of IdU-labelled cells was evaluated in corneas and limbus after 15 min (n = 6) or 2 h (n = 6) the labelling rate plateaued, suggesting all cells in S-phase were labelled within 15 min and proportionately few new cells entered S-phase in 2 h. Limbal epithelia had higher mean (%) ± SEM of proliferating IdU-labelled cells (15 min, 11.3 ± 1.6; 2 h, 11.4 ± 2.1 respectively) than in corneal epithelia (7.0 ± 0.9; 7.1 ± 0.9, respectively) (Fig. 3B). Two-way analysis of variance (ANOVA) followed by pairwise Bonferroni post-hoc tests showed a nonsignificant main effect of IdU-circulation time on selected tissues: corneal and limbal epithelia; F (2, 26) = 0.2, p = 0.86. However, it showed IdU-labelled cells were present in significantly higher numbers in the limbal than corneal epithelia; F (1, 26) = 12.9, p = 0.001. It was concluded that proportionately more cells are in S-phase at any one time in the limbal epithelium than in the basal corneal epithelium. Most of these dividing cells are presumed to be rapidly dividing transit amplifying cells (TACs), derived from limbal epithelial stem cells (LESCs), and consistent with a model of the limbal progenitors being more proliferative than those of the central cornea.

### 3.3 Cycling kinetics of proliferating cells in corneal and limbal epithelia

**Fig. 4:**
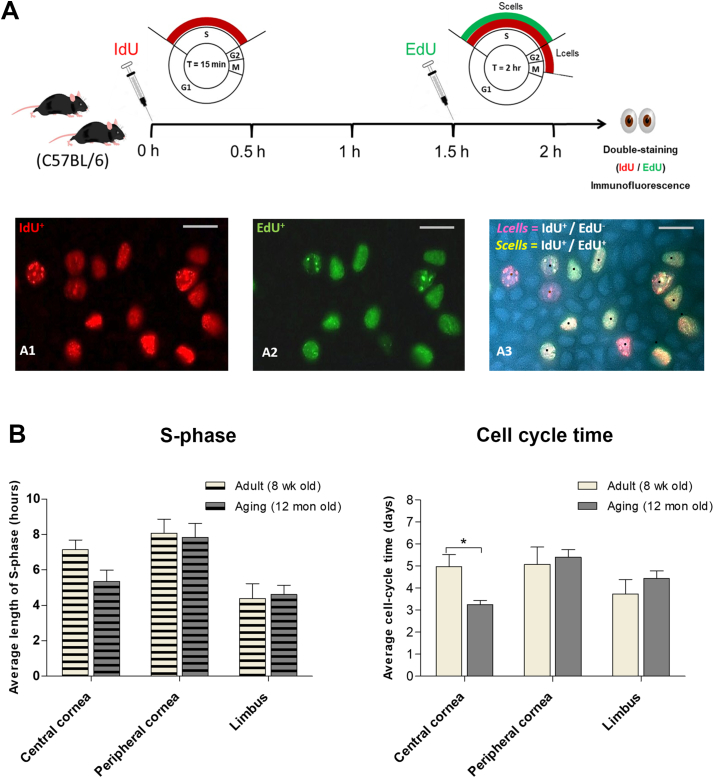
Cell cycle kinetics in corneal and limbal epithelia of mice. (A) Methodological scheme illustrating the 1.5-h interval between IdU and EdU injections in vivo. IdU-labelled cells red (A1), EdU-labelled green (A2). The red-only cells in (A3) marked with brown dots represent the *L*_cells_. The red and green double-labelled cells in (A3) represent the *S*_cells_. Scale bars = 10 μm. (B) Average S-phase (*Ts*) and cell cycle time (*Tc*) (mean ± SEM) in epithelia of central cornea, peripheral cornea and limbus of 8 week and 12 month-old C57BL/6 mice. P < 0.05 (*t*-test; n = 6) is shown by the asterisk (*). (For interpretation of the references to colour in this figure legend, the reader is referred to the web version of this article.)

In order to evaluate the kinetics of transient amplifying cells (TACs) in corneas of adult mice during homeostatic conditions, a short-term dual-pulse labelling technique was used. Adult mice either 8 weeks or 12 months old on a C57BL/6 genetic background were used, with each mouse receiving a single injection of IdU followed 1.5 h later by a single injection of EdU for 30 min before mice were killed and anti IdU/EdU immunohistochemistry and staining was performed. The number of single-labelled and double-labelled cells was counted separately in central cornea, peripheral cornea and limbal epithelia. From these values, length of S-phase (*Ts*) and cell cycle time (*Tc*) were estimated using the formulae described in the Methods section and are presented in Table 1 and Fig. 4. In all regions of the cornea/limbus, cell cycle time was calculated to be around 3–5 days, consistent with rapid turnover of basal cells. No significant difference was found between estimated length of S-phase or cell cycle time in the limbal or peripheral corneal epithelia of 12 month old mice compared to the young adults. Turnover of the central cornea of aging mice (*Tc* 3.24 ± 0.2 days) was however significantly faster than that of the 8-week old mice (4.97 ± 0.5 days) (P < 0.05). These data indicated that aging may not affect the proliferation kinetics of the limbal epithelium, at least in a short-term assay that captures the activity of rapidly proliferating, transit amplifying cells. The estimates of cell cycle time rest on the assumption that all cells in the population are cycling (*P*_cells_) – whereas this assumption is probably true for the corneal epithelium, in the limbus the known presence of slow cycling or quiescent stem cells means that the true cell cycle time of limbal TACs has probably been overestimated – this is discussed below.

**Table 1:** Length of S-phase (Ts) and cell cycle (Tc) of limbal and corneal epithelia.

Background	Age	*Ts* (hours)			*Tc* (days)		
		Limbus	Peripheral cornea	Central cornea	Limbus	Peripheral cornea	Central cornea
C57BL/6	8 week	4.39 ± 0.83 (n = 6)	8.06 ± 0.80 (n = 5)	7.14 ± 0.54 (n = 6)	3.73 ± 0.65 (n = 6)	5.07 ± 0.78 (n = 5)	4.97 ± 0.5 (n = 6)
C57BL/6	12 month	4.62 ± 0.51 (n = 6)	7.84 ± 0.78 (n = 5)	5.34 ± 0.64 (n = 6)	4.43 ± 0.35 (n = 6)	5.40 ± 0.35 (n = 5)	3.24 ± 0.2 (n = 6)

### 3.4 Slow-cycling LESCs are identified as label-retaining cells

**Fig. 5:**
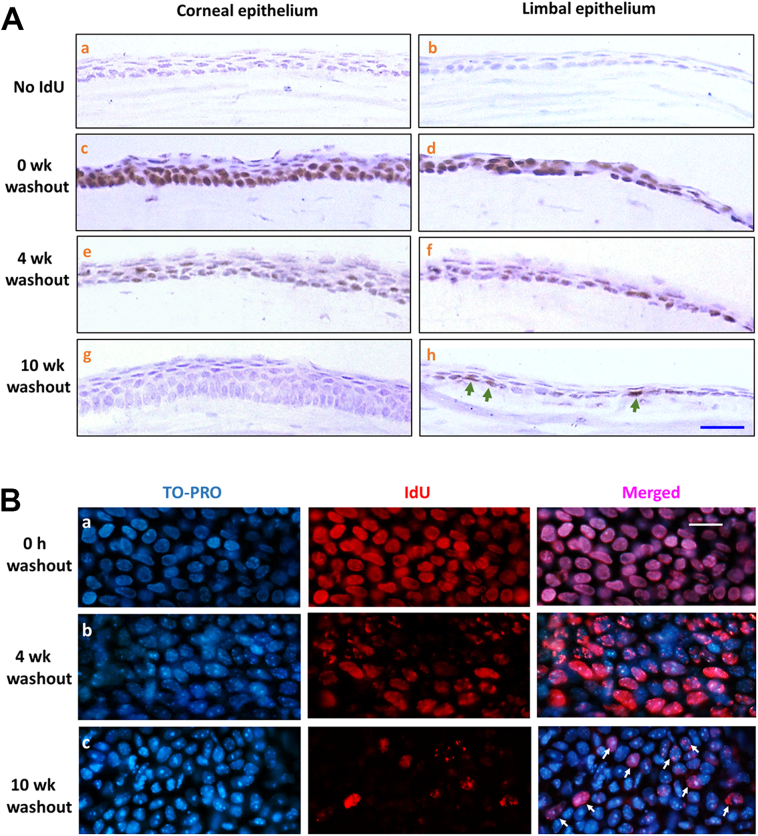
Location of IdU Label-retaining cells. Corneal and limbal tissues from adult (C57BL/6) mice exposed to long-term IdU treatment (30 days) followed by washout periods: 0 week, 4 weeks and 10 weeks. (A) Hematoxylin-stained cross-sections of corneal and limbal tissues. IdU visualized by immunostaining (brown). (a, b) No IdU-treatment (negative controls). (c, d) 0-week washout – all cells labelled. (e, f) 4-weeks washout. (g,h) IdU-label undetectable in the corneal epithelium after 10-weeks washout but ~25% of basal limbal epithelial cells retain IdU-label (green arrows). Scale bar = 20 μm. (B) IdU-immunostaining (red) of flat-mounted limbal epithelia after 30 days IdU labelling. TO-PRO3 (blue) nuclear stain. (a) 0-week washout shows IdU label in 100% of the cells. (b) 4-weeks washout. (c) 10-weeks washout period reveals ‘label-retaining cells’ indicated by white arrows. Scale bar = 10 μm. Abbreviations: wk., week; h, hours.

Because LESCs are believed to divide infrequently under steady-state condition in adult mice, they can be visualized as label-retaining cells (LRCs) as described above. Long-term (30 days) exposure of 8 to 12-week old C57BL/6 mice to IdU in drinking water was performed followed by washout periods of 0–10 weeks to select the washout period required to visualise retained IdU label only in slow-cycling limbal cells and not in the corneal epithelium. Histological staining (Fig. 5A) revealed all cells to be IdU-labelled in the corneal and limbal epithelia immediately after the end of the labelling period. After 4 weeks of washout (estimated 5–7 cell cycles based on data in Fig. 4), fewer label-retaining cells were detected in the corneal epithelium, as it was diluted out in rapidly dividing cells, and by 6 weeks only a few superficial corneal epithelial cells had detectable label. After 10 weeks of washout, no label-retaining cells remained in the corneal epithelium, but IdU-LRCs were clearly identified in limbal epithelium. Similarly, IdU immunofluorescent staining on whole-mount corneas revealed IdU-labelled cells in limbal epithelia (Fig. 5B) after different interval of washout periods: 0 week, 4 weeks, and 10 weeks. The 0-week washout showed most limbal cells labelled with IdU. By 10-weeks washout, the fluorescence labelling revealed approximately 23% of limbal cells with a variable but speckled pattern of IdU retention consistent with dilution over 3–4 cell cycles and approximated to a cell division every 2–3 weeks. These represent the active, but slowly dividing, LESCs, expressing stem cell markers such as Sox9 and ΔN-P63α, consistent with previous studies (Lehrer et al., 1998; Pajoohesh-Ganji et al., 2006; Sartaj et al., 2017; Zhao et al., 2009; Douvaras et al., 2013). It was concluded therefore, that a minimum 10–11 week washout period (equivalent to at least 14 cell cycles for rapidly dividing cells according to estimates above, and at least three complete turnovers of the central corneal epithelium (Douvaras et al., 2013), was sufficient to conclude that any labelled cells were slow cycling, ‘label-retaining’ cells.

### 3.5 The response of IdU-label-retaining cells in limbus to corneal epithelial wound in adult and aging mice

**Fig. 6:**
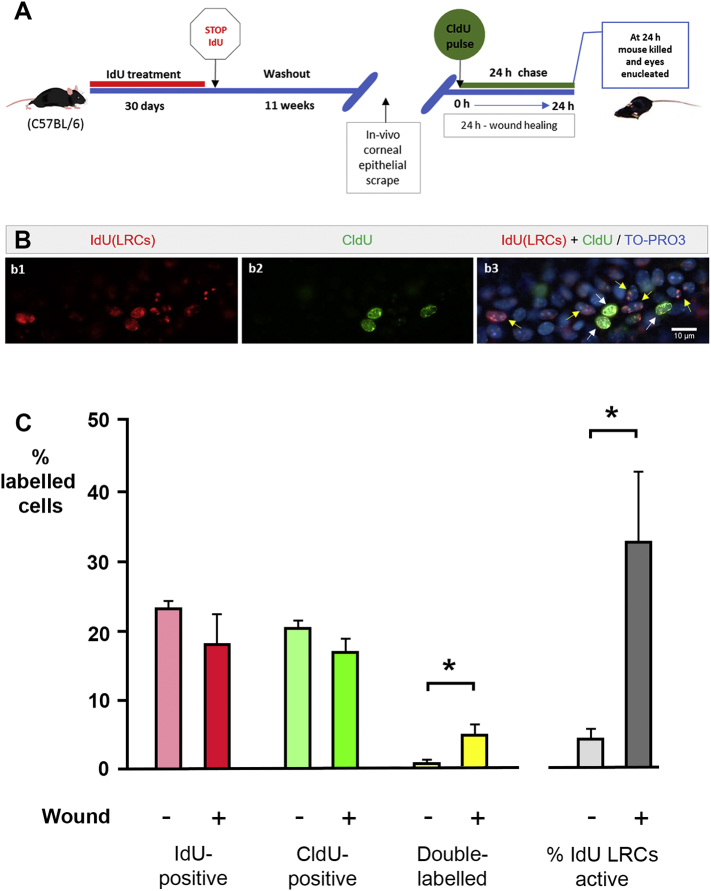
Activity of label-retaining cells in limbal epithelia 0–24 h after corneal wounding. (A) Schematic representation of a pulse-chase-pulse experimental design illustrating the exposure of 8 week-old C57BL/6 mice to 30 days IdU treatment, subsequent 11-weeks washout, CldU injection at 0 h after unilateral corneal epithelial wound in vivo, and mice death at 24 h. (B) Immunofluorescent images of proliferative cells in flat-mounted idU-labelled limbal epithelium after wounding and CldU pulse: (b1) IdU (red); (b2) CldU (green); (b3) Merged image including TO-PRO3-label (blue). Double-labelled cell indicated by white arrows are actively proliferative label-retaining cells. Yellow arrows indicate IdU-positive, CldU-negative (stem) cells that did not divide after wounding. (C) Cell proliferation in the limbal epithelia of unwounded and wounded corneas. Data are expressed as mean ± SEM of: %IdU-positive cells as a proportion of *all* cells in limbal epithelium – pink/red), %CldU-positive cells (green), and % of double-labelled cells (yellow) 24 h after wounding. Grey bars show the % of double-labelled cells as a proportion of the IdU-positive cells and represents the percentage of label-retaining stem cells that were dividing in wounded and unwounded eyes. There was a significant sevenfold increase in the percentage of label-retaining stem cells entering mitosis in wounded eyes (P < 0.05 (*), Mann–Whitney *U* test, n = 6 wounded, 6 unwounded. (For interpretation of the references to colour in this figure legend, the reader is referred to the web version of this article.)

To study the proliferative ability of IdU-LRCs in the limbal epithelia, 8-week old wild-type C57BL/6 mice were IdU-labelled for 30 days via drinking water, followed by 11-week washout. They were then given a unilateral corneal epithelial scrape wound as described in Materials and Methods, injected with a single dose of CldU at 0 h after wounding (Fig. 6A) and killed 24 h later. After immunohistochemistry, cells were identified in the limbal epithelium that had retained ‘speckled’ patterns of IdU labelling, consistent with a slow cycling nature, having undergone 5 cell divisions or fewer during the washout period. The CldU injection identified cells that were dividing in the period immediately after wounding. Double-labelled, IdU^+^/CldU^+^ cells were those label-retaining cells, presumed stem cells that were mitotically active in the 24 period after wounding, in wounded and unwounded eyes. Indicative basal levels of labelling in control mice with no wounding in either eye at time of death were 24.5 ± 1.2% IdU label-retaining cells in the limbus, with 3.0 ± 1.1% of label-retaining cells double-labelled with CldU (hence in active proliferation at any one time) (n = 3). Cells in wounded eyes and contralateral unwounded eyes were scored as labelled with IdU-only (‘IdU-LRCs’), labelled with CldU only, double-labelled or unlabelled. 0–24 h after surgery, IdU-LRCs represented 23.4 ± 0.8% of cells in limbal epithelia of unwounded corneas, higher than in wounded (18.0 ± 4.3%). The difference was nonsignificant (P = 0.251, paired Student's *t*-test, n = 6) (Table 2; Fig. 6B) but the downward trend would be consistent with proliferation of label-retaining stem cells increasing after wounding diluting out the IdU to an undetectable level in some cells. The proportion of IdU-retaining cells that were also double-labelled with CldU (i.e. the stem cells in active proliferation) was found to be significantly higher in wounded corneas (32.8 ± 10.0%) than in unwounded corneas (4.52 ± 1.4%) (P = 0.036), Mann–Whitney *U* test, n = 6) (grey bars in Fig. 6, Table 2). That result demonstrates around an additional seven-fold increase in LESC proliferation after wounding of the cornea, which is the first time this has been quantified.

**Table 2:** Label-retaining (stem) cells detected and proportion active in wounded and unwounded eyes.

Age at start of experiment	Age at end of experiment	Treatment	Wound	N	%IdU-LRCs	%CldU or EdU positive cells	% Double-labelled cells	% of IdU-LRCs active
8 week	23 week	CldU at 0–24 h post-wounding	No	6	23. 35 ± 0.84	20.39 ± 1.16	1.01 ± 0.31	4.5 ± 1.4
8 week	23 week	CldU at 0–24 h post-wounding	Yes	6	18.02 ± 4.33	16.91 ± 1.86	5.12 ± 1.41	32.8 ± 10.0
37 week	52 week	CldU at 0–24 h post-wounding	No	4	22.06 ± 2.43	17.95 ± 3.16	2.51 ± 1.06	15.9 ± 6.9
37 week	52 week	CldU at 0–24 h post-wounding	Yes	4	25.01 ± 4.32	12.73 ± 0.58	3.53 ± 1.48	12.2 ± 5.6
8 week	23 week	EdU at 24–48 h post-wounding	No	12	8.79 ± 1.37	20.05 ± 2.03	3.41 ± 0.75	46.0 ± 9.44
8 week	23 week	EdU at 24–48 h post-wounding	Yes	12	11.39 ± 2.37	32.71 ± 1.92	10.82 ± 2.37	86.5 ± 8.16

**Fig. 7:**
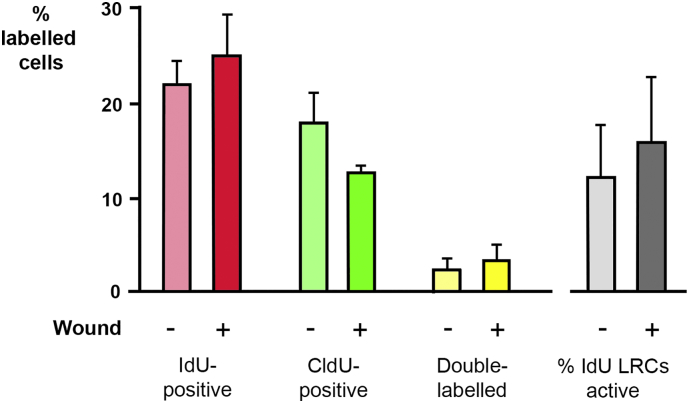
Activity of label-retaining cells in limbal epithelia 0–24 h after corneal wounding in aging mice. Cell proliferation in the limbal epithelia of unwounded and wounded corneas in old mice (1 year). Data are expressed as mean ± SEM of: % IdU-positive cells as a proportion of *all* cells in limbal epithelium – pink/red), % CldU-positive cells (green), and % of double-labelled cells (yellow) 24 h after wounding. Grey bars show the % of double-labelled cells as a proportion of the IdU-positive cells and represents the percentage of label-retaining stem cells that were dividing in wounded and unwounded eyes. There were no significant differences between wounded and unwounded corneas. (For interpretation of the references to colour in this figure legend, the reader is referred to the web version of this article.)

The impact of corneal aging on the activity and proliferative ability of IdU-LRCs in limbal epithelium was assessed in aging C57BL/6 mice. The experiment above was repeated on mice aged 8 months old at start of labelling period, rather than 8 weeks (Fig. 7A). For these mice the mean percentage ± SEM of IdU-LRCs between unwounded and wounded corneas was not significantly different (22.06 ± 2.43% and 25.01 ± 4.32%, respectively) and, in contrast to the data for younger mice (Fig. 6), the mean percentage of double-labelled cells as a proportion of the total number of cells retaining IdU did not significantly increase in wounded corneas when compared to unwounded (15.9 ± 6.90% and 12.2 ± 5.6%, respectively) (Table 2; Fig. 7B). These numbers indicate that IdU-LRCs in aging corneal tissues did not become increasingly active in response to wounding.

### 3.6 Systemic activation of stem cells 24–48 h after wounding

**Fig. 8:**
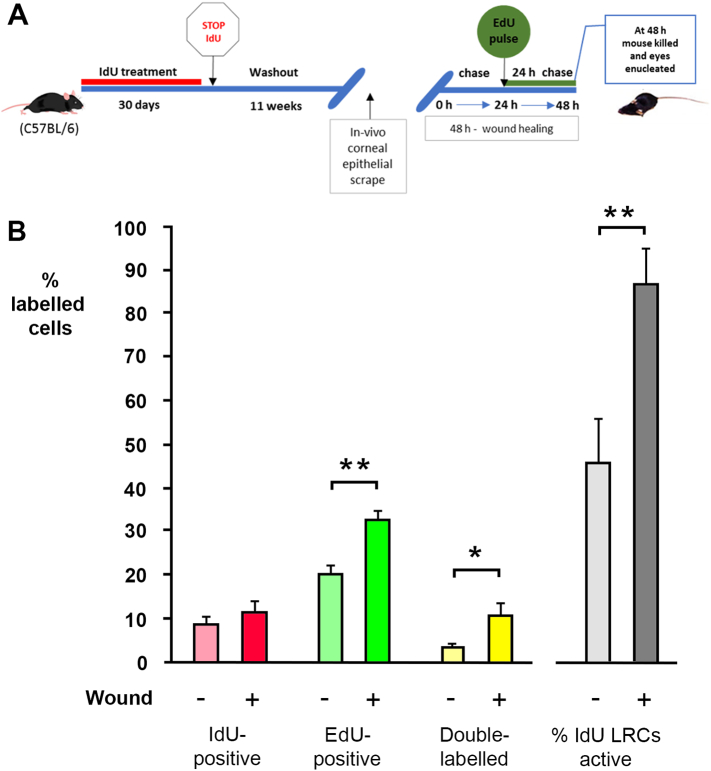
Activity of label-retaining cells in limbal epithelia 24–48 h after corneal wounding in adult mice. (A) Schematic representation of a pulse-chase-pulse experimental design illustrating the exposure of 8 weeks old C57BL/6 mice to 30 days IdU treatment, 11-weeks washout and EdU pulse 24 h after unilateral corneal epithelial wounding with mice death at 48 h. (B) Cell proliferation in the limbal epithelia of unwounded (n = 12) and wounded (n = 12) corneas. Data are expressed as mean (%) ± SEM. The mean percentage of EdU-labelled cells increased significantly in wounded corneas compared to unwounded (P < 0.05 (**), *t*-test, n = 12). There was also a significant increase in the number of double-labelled cells (P < 0.05 (*), *t*-test, n = 12) both in absolute terms and as a proportion of the total number of label-retaining cells 48 h after wounding.

We were able to detect activation of limbal stem cell proliferation in C57BL/6 mice in the 24 h immediately after wounding, which represents the phase of initial re-epithelialisation. Wounds had generally healed after 24 h but it remained possible that elevated stem cell activity would remain detectable in the next 24-hour period when stratification of the healed epithelium continues. This was tested. The long-term labelling and wounding experiment was repeated on 8-week old mice but the proliferative activity of IdU-label-retaining cells was determined 24–48 h after wounding, using a single injection of EdU at 24 h after wounding (Fig. 8A), followed by analysis at 48 h. In wounded eyes, the percentage of IdU-retaining cells dropped further (compared to 0–24 h, above) to 11.39 ± 2.369%, with an increase in the proportion of double labelled cells (86.5 ± 8.16%) indicating most label-retaining stem cells had entered the cell cycle since wounding (Fig. 8; Table 2). Surprising however, the data for the contralateral unwounded eyes indicated 46.0 ± 9.44% of IdU-positive label-retaining cells were also double-labelled with EdU (a tenfold increase over that seen in unwounded eyes at 0–24 h) (Fig. 8; Table 2). These data suggested that, after a lag of around 24 h, wounding to one cornea activates limbal stem activity in both eyes – a previously unsuspected systemic effect that is discussed below.

## 4 Discussion

### 4.1 Cycling kinetics of ocular surface epithelia

This study used thymidine analogue double-labelling to define basic parameters of limbal and corneal epithelial cell proliferation during normal homeostasis and during aging. The administration of 1 mg/ml IdU via drinking water was shown to be a safe and effective means of delivery of DNA label over extended periods to adult mice, and avoids the use of osmotic minipumps or repeated injections. In combination with CldU and EdU, it was shown to be possible to achieve specific flat-mount two-colour fluorescence labelling of tissues, and therefore to estimate cell cycle parameters and the activation of label-retaining, putative stem cells, in vivo. This allowed us to revisit and extend some of the basic assumptions regarding corneal maintenance and stem cell biology, essential to clinical understanding of problems associated with injury and aging.

### 4.2 Cell cycle time in ocular surface epithelia

No study has addressed how long it takes for a DNA-labelling reagent such as IdU to reach the ocular surface. We found that labelled cells could be detected in the limbal epithelium within 5 min of IdU injection. Given that we subsequently calculated S-phase in limbal epithelia in those mice to be of the order of 4 h (Table 1), this suggests that as little as 2% genomic incorporation of IdU is detectable (defining 100% incorporation as that taken up during a full S-phase). Were a cell to incorporate IdU under these conditions for a full S-phase, it would therefore require 5–6 cell cycles of washout for IdU to become undetectable again. This corresponds well with our observations of washout in vivo and in vitro (Fig. 2).

Furthermore, the cell cycle parameters of TACs were measured at three sections of corneal epithelium: central cornea, peripheral cornea, and limbus. We found no evidence for the hypothesis that cell cycle time lengthens as the basal corneal epithelial cells reach the end of their proliferative ‘life’ (Table 1; Fig. 4). Cells in the peripheral and central corneal epithelia of 8 week old C57BL/6 mice all had mean cell cycle time in the region of 4–5 days.

Estimation of cell cycle time using the formula described in Martynoga et al. (2005) requires an assumption that all the cells counted (*P*_cells_) were actively mitotic. This is probably true for basal corneal epithelial cells - a single injection of IdU labels about 7% of cells in the corneal epithelium (Fig. 3), inferring S-phase requires about 7% of total cell cycle time, a good match for estimates using the formula (Fig. 4; Table 1), suggesting all cells are actively mitotic. However, for the limbus, the fact that a single injection of IdU labels around 11% of cells, but our calculations (Fig. 4; Table 1) infer S-phase is only 5% of cell cycle time, suggests the number of *P*_cells_ has been overestimated and that many limbal cells are not actively cycling at any given time. Our subsequent investigations suggested at least 20–25% of the basal limbal epithelial cells were label-retaining, presumed LESCs, that are unlikely to be in active division during the short-term labelling experiment, mean cell cycle time of the rapidly dividing limbal epithelial TACs are likely to be about 20–25% lower than the estimates in Table 1.

An earlier study showed that some basal epithelial cells located in unwounded peripheral corneas may undergo two rounds of cell division within 60–72 h (Lehrer et al., 1998), faster than the cell cycle time estimated in this study. The discrepancy may be explicable in that the previous study identified the fastest cycling cells whereas our estimate is a mean of the entire population.

### 4.3 Label-retaining stem cells in the basal limbal epithelium

Previous studies have shown that the exposure of corneal tissues to stimulatory factors such as tumour promoter 12-O-tetra-decanoylphorbol-13-acetate (TPA) or injury (Cotsarelis et al., 1989; Lehrer et al., 1998); or an antimetabolite, fluorouracil (5-FU), (Tseng and Zhang, 1992), was accompanied by proliferation of cells in the limbal epithelium within 48 h. Nevertheless, the use of tritiated thymidine in those studies did not allow whole-mount analysis of labelling patterns and quantitative data were not fully presented. Our data are consistent with but significantly advance previous work:

1) True ‘label-retaining’ cells are present normally in the limbal but not corneal epithelium. We used an 11 week washout period, similar to Douvaras et al. (2013) and longer than the 6 week washouts used previously (Lehrer et al., 1998; Zhao et al., 2009). Other studies that report label-retaining cells within the corneal epithelium used a shorter washout (4 weeks) and may therefore have detected BrdU remaining in transit amplifying cells (Haddad and Faria-E-Sousa, 2014; Li et al., 2017).

The data presented in this study show, in addition, that:

2) In adult mice at least 20% of basal limbal epithelial cells are slow cycling, presumed LESCs. Patterns of label loss suggest they may normally divide every 2–3 weeks on average.
3) There is a statistically significant activation of proliferation in the limbal epithelial stem cells of wounded corneas within 24 h, eight times that seen in the contralateral eyes and in unwounded mice (thus supporting the model of limbal stem cell action in response to injury).
4) However, during the 24–48 h period after wounding, there is a continued and increased level of activation of stem cells in both wounded and contralateral unwounded eyes. This is an unexpected result that points to a systemic stem cell activation following localized injury.

### 4.4 How do slow-cycling LESCs in adult corneas respond to injury?

When the corneal epithelium is injured, calcium release from lesioned cells initiates neighbouring intact cells to release calcium from intracellular stores, leading to a propagating wave of calcium signaling from the wound edge in 2–3 min (Leiper et al., 2006). This in turn activates secondary messengers such as MAP-kinase cascades within about 30 min (Leiper et al., 2006). Cell migration to fill the wound starts within 2–6 h in vivo, and elevated levels of corneal epithelial mitosis after about 6 h (Walczysko et al., 2016). Our data show that at least a quarter of LESCs must also be induced to undergo additional mitoses within a few hours of a large wound, presumably activated by the same growth factor signaling that induces the physiological wound healing change of the corneal cells. Our data however show that induction of LESC mitosis is progressive. 48 h after wounding, and >24 h after initial re-epithelialisation is complete, 80–90% of LESCs could be shown to be in active S-phase in wounded eyes. Whereas at 0–24 h after wounding, contralateral unwounded eyes showed levels of label-retention and stem cell activity similar to that seen in mice that had received no injury, proliferation increased significantly in the 24–48 h period. Wound-induced release of cytokines and growth factors such as hepatocyte growth factor and epidermal growth factor into the vasculature (Yu et al., 2010) is also likely to be progressive, and could explain both the prolonged response of wounded eyes and concomitant increase of mitotic activity in contralateral unwounded eyes. Neurotransmission and the release of neurotrophins from corneal epithelial nerves damaged by injury may also play a part in the progressive wound healing response. Cell bodies of the trigeminal ganglion synapse in the trigeminal nucleus of the brainstem, from where signals are transmitted to the thalamus and primary somatosensory cortex (Rosenthal et al., 2016), suggesting a possible mechanism of neural transmission of the wound healing response to the contralateral eye.

It would be useful to elucidate what potential benefit there could be for the contralateral eye to elicit a wounding response. Acute eye injuries of the type performed in this paper are perhaps not common in nature and when they occur, may be unlikely to affect both eyes. However, the types of chronic physical or other insults due to dust, noxious substances, levels of ultraviolet exposure and infectious disease, that may stimulate LESC activity, are more likely to affect both eyes at once, so there may be some selective advantage for both eyes to coordinate a wounding response. It is also possible that the activation of contralateral LESCs following wounding is neutral to fitness and just consequence of the systemic release of wound-induced growth factors. Further studies could resolve this: for example if stem cells unrelated to ocular surface injury in the skin epidermis were also shown to be activated.

### 4.5 Limbal epithelial stem cell quiescence

It is not to be expected that the LESCs all show equal levels of activity at any one time. Li and Clevers (2010) proposed from studies conducted in tissues such as hair follicle and bone marrow that there is reciprocal ‘backup zone’ between quiescent and active stem cells, governed by inhibitory and stimulatory signals. ‘Active’ stem cells may be primed for tissue regeneration and repair, whereas ‘quiescent’ cells are reserved cells that act as a backup to replace damaged or lost active stem cells. The presence of quiescent LESCs was recently suggested through a lineage tracing study that suggested LESCs have phases of quiescence and activity (Dorà et al., 2015). LESCs that were completely quiescent during the labelling period would be missed by our assays. The discrepancy between the number of limbal epithelial cells labelled by a single IdU injection (Fig. 3) and our subsequent calculation of S-phase and cell cycle time (Fig. 4; Table 1), described above, is explicable if in addition to the 20–25% of slow-cycling label-retaining cells, another 20–25% of limbal epithelial cells are quiescent, non-mitotic over the labelling period – limbal ‘dark matter’.

### 4.6 The aging ocular surface

In general, the cornea is remarkably resistant to aging, with little or no loss of transparency or other pathology routinely occurring in the elderly. However, many structural and functional changes in corneas have found to be associated with rise in age (Faragher et al., 1997). For instance, the corneal epithelial surface becomes smoother because of the loss of microvilli, microplicae, and glycocalyx (Eckard et al., 2006) and the thickness of the peripheral corneal and limbal epithelia (nasal and temporal quadrants) decreases, whereas central corneal thickness is unchanged (Yang et al., 2014). Most corneal aging research has studied endothelial degeneration, as the density of non-replicative endothelial cells reduces with advancing age (Oh, 1963; Fitch et al., 1982; Murphy et al., 1984). How aging affects LESCs is not known, although cell density in the human basal limbal epithelia decreases with increasing age (Patel et al., 2006; Niederer et al., 2007). Corneal aging studies in human identified a reduction in the presence of palisades of Vogt (the niche for stem cells) and limbal crypts with increasing age (Zheng and Xu, 2008; Notara et al., 2012). However, the mouse limbus lacks the anatomical specialisation of the human limbus. Lineage tracing studies in mice have shown a reduction in the numbers of coherent clones of LESCs with age, from about 100 per eye in adult (10 weeks) to about 50 in aging mice (39 weeks) (Collinson et al., 2002; Mort et al., 2009; Amitai-Lange et al., 2015), however this does not necessarily imply a loss of stem cells and can be explained by neutral drift in clone size (West et al., 2018).

This study provided preliminary evidence of some changes to the proliferative capacity of basal epithelial cells in the ocular surface with aging (mice 11–12 months old). At this stage, mice are past normal breeding age and mortality increases rapidly, however their corneas were transparent and overtly normal. When wounded, although healing rate was not measured directly, the corneas re-epithelialised normally overnight. As for younger mice, about 20–25% of cells in the basal limbal epithelia were label-retaining, presumed stem cells. However, wounding did not cause any increase in proliferation rate compared to unwounded contralateral eyes, suggesting a possible attenuation of their ability to respond to acute corneal wounding (Table 2; Fig. 7). Mean cell cycle time of rapidly dividing TACs in the basal corneal and limbal epithelia of 12-month old mice were similar to those of 8-week mice, but in the central corneal epithelium, mean cell cycle time decreased significantly to about 3.25 days (Table 1). These data suggesting that central cornea of aging eyes may need to increase cycling, possibly to compensate for epithelial fragility of decreased sensitivity of the stem cells to injury. Further work will be needed to determine whether he LESCs or aging mice show any response in the 24–48 period after wounding.
